# Analysis of Breast Cancer Family History, Estrogen Receptor Status, and Breast Cancer Outcomes in Sweden

**DOI:** 10.1001/jamanetworkopen.2023.18053

**Published:** 2023-06-13

**Authors:** Yuqi Zhang, Qiao-Li Wang, Erwei Zeng, Wei He, Kamila Czene

**Affiliations:** 1 Department of Medical Epidemiology and Biostatistics, Karolinska Institutet, Stockholm, Sweden; 2 Department of Medical Oncology, Dana-Farber Cancer Institute and Harvard Medical School, Boston, Massachusetts; 3 Chronic Disease Research Institute, The Children’s Hospital, National Clinical Research Center for Child Health, School of Public Health, School of Medicine, Zhejiang University, Hangzhou, China

## Abstract

**Importance:**

Breast cancer (BC), the most prevalent cancer among women globally, is a heterogeneous disease, with prognosis differing by estrogen receptor (ER) status. Having a family history of BC increases the risk of BC; however, it is unclear whether family history is associated with the prognosis of overall and ER-specific BC.

**Objective:**

To assess whether a family history of BC is associated with the prognosis of overall and ER-specific BC.

**Design, Setting, and Participants:**

This cohort study was based on data from several national registers in Sweden. All female residents of Stockholm who were born after 1932; had their first BC diagnosis between January 1, 1991, and December 31, 2019; and had at least 1 identified female first-degree relative (FDR) were included. Women who were diagnosed with other types of cancer before their BC diagnosis, were older than 75 years at diagnosis, or had distant metastasis at diagnosis were excluded. A total of 28 649 women were included. Data were analyzed from January 10, 2022, to December 20, 2022.

**Exposures:**

Family history of BC, defined as 1 or more female FDRs diagnosed with BC.

**Main Outcomes and Measures:**

Patients were followed up until BC-specific death, censoring, or end of follow-up on December 31, 2019. The role of family history in BC-specific mortality was investigated using flexible parametric survival models among the full cohort, ER-positive subgroup, and ER-negative subgroup, adjusting for demographic characteristics, tumor characteristics, and treatments received.

**Results:**

Among 28 649 patients, the mean (SD) age at BC diagnosis was 55.7 (10.4) years; 19 545 (68.2%) had ER-positive BC, and 4078 (14.2%) had ER-negative BC. Overall, 5081 patients (17.7%) had at least 1 female FDR diagnosed with BC, while 384 (1.3%) had a family history of early-onset BC (FDR diagnosed before age 40 years). During the follow-up period (median [IQR], 8.7 [4.1-15.1] years), 2748 patients (9.6%) died of BC. Multivariable analyses revealed that having a family history of BC was associated with a lower risk of BC-specific death among the full cohort (hazard ratio [HR], 0.78; 95% CI, 0.65-0.95) and the ER-negative subgroup (HR, 0.57; 95% CI, 0.40-0.82) in the first 5 years, after which no association was observed. However, having an early-onset family history was associated with a higher risk of BC-specific death (HR, 1.41; 95% CI, 1.03-2.34).

**Conclusions and Relevance:**

In this study, patients with a family history of BC did not necessarily have a worse prognosis. Those with ER-negative status and a family history of BC had more favorable outcomes in the first 5 years after diagnosis, possibly due to enhanced motivation to receive and adhere to treatment. However, patients with a family history of early-onset BC had worse survival, suggesting that genetic testing of newly diagnosed patients with early-onset family history may provide useful information to aid treatment and future research.

## Key Points

**Question:**

Is a family history of breast cancer (BC) associated with the prognosis of overall and estrogen receptor–specific BC?

**Findings:**

In this cohort study of 28 649 female patients with BC in Sweden with up to 15 years of follow-up, having a family history of BC was associated with a lower risk of BC-specific death. This association was limited to the first 5 years after diagnosis and the estrogen receptor–negative subgroup and was not likely due to screening-associated early detection.

**Meaning:**

These findings suggest that patients with a family history of BC do not necessarily have worse outcomes, and some patients may have better outcomes, possibly due to enhanced motivation to receive and adhere to treatment.

## Teaser

This study uses data from national registers to assess the association of family history of breast cancer with overall and estrogen receptor–specific breast cancer outcomes among women in Sweden.

## Introduction

Breast cancer (BC) is the most prevalent cancer, with a wide spectrum of clinical and pathological presentations.^1^ One of the most important features to characterize BC is estrogen receptor (ER) status, according to which BC is categorized into ER positive and ER negative.^2^ Patients with ER-negative BC are typically younger, insensitive to hormonal therapy, and have a lower 5-year survival of 78% compared with 89% in patients with ER-positive BC.

Family history of BC is a well-established risk factor for BC; having 1 first-degree relative (FDR) with BC is associated with 1.8-fold higher risk, and having 2 FDRs with BC is associated with 2.9-fold higher risk.^3^ Whether family history should be a concern in patient prognosis has been a subject of research interest. A systematic review^4^ published in 1999 summarized evidence on the association between family history of BC and BC prognosis and concluded that conflicting data existed, mostly due to small samples. Since then, studies with larger samples^5,6,7,8,9,10,11,12,13,14,15^ have been conducted; however, these studies had mixed findings, with some reporting worse prognosis among those with a family history of BC,^5,6,7^ some finding no significant difference,^8,9,10,11,12^ and some reporting that family history had a protective benefit for prognosis.^13,14,15^ Even less is known about whether the association differs across BC subtypes, such as ER-positive and ER-negative BC.

Early-onset BC has been a special group in terms of biological and histopathological characteristics, with a higher predisposition to certain genetic variants such as in *BRCA*, and has been consistently reported to have a worse prognosis.^16,17,18,19,20,21,22^ Family history of early-onset BC has been associated with a significant increase in a healthy woman’s risk of BC, with 3.9- to 13.5-fold higher risk among those with a female FDR who received a BC diagnosis before age 40 years.^3^ However, it is less clear how such a family history is associated with BC-specific survival.

Thus, the primary aim of this cohort study was to assess whether family history of BC was associated with prognosis of overall and ER-specific BC among a large and well-characterized Swedish cohort of patients with BC. The secondary aim was to further investigate whether this association differed when family history of BC was disaggregated into early onset and non–early onset.

## Methods

### Study Population

This cohort study was approved by the ethical committee of Karolinska Institutet with a waiver of informed consent due to the use of deidentified data. The study followed the Strengthening the Reporting of Observational Studies in Epidemiology (STROBE) reporting guideline for cohort studies.^23^

**Figure 1.**
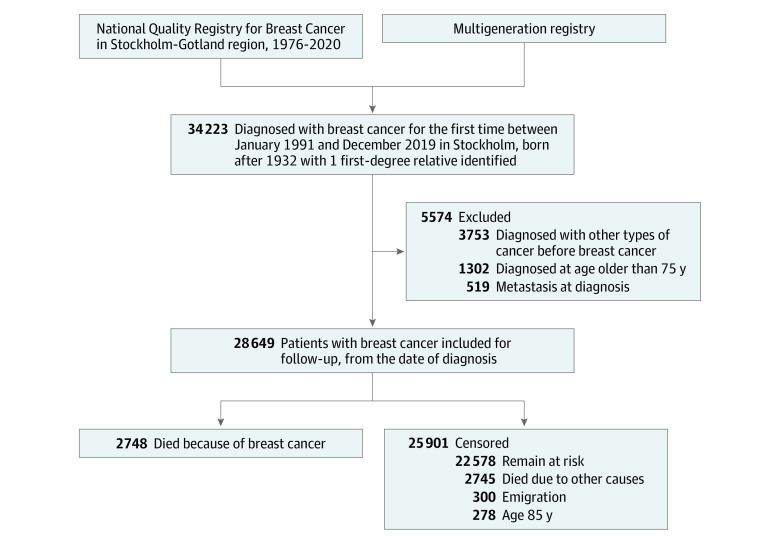
Flowchart of Study Enrollment and Follow-up

This study selected a patient cohort based on data from Swedish national registers using unique personal identification numbers. All women born after 1932 and diagnosed with BC in Stockholm were identified through the Stockholm-Gotland Quality Register for Breast Cancer (comprising data from the Stockholm-Gotland Breast Cancer Register [1976-2007] and the National Quality Register for Breast Cancer [2008 to present]).^24,25^ In addition, to obtain information on family history of BC, we only included women with at least 1 female FDR identified through the Swedish Multi-Generation Register.^26^ The flowchart of enrollment of the study population, including inclusion and exclusion criteria, is shown in Figure 1. Briefly, we included 34 223 women with a first diagnosis of BC between January 1, 1991, and December 31, 2019, who had at least 1 identified female FDR. The baseline of January 1, 1991, was chosen to ensure the accuracy of family history; women alive on January 1, 1991, have complete parental information in the Swedish Multi-Generation Register.^26^ We excluded 5574 women who (1) were diagnosed with other types of cancer before BC (n = 3753), (2) were older than 75 years at diagnosis (n = 1302), or (3) had distant metastasis at diagnosis (n = 519). The remaining 28 649 women were included in the study. We then followed up the cohort from diagnosis until the outcome of interest, censoring, or end of the follow-up period on December 31, 2019. Data were analyzed from January 10, 2022, to December 20, 2022.

### Baseline Characteristics

Baseline patient characteristics included age at diagnosis and menopausal status. Tumor characteristics included tumor size, grade, and lymph node status. Molecular characteristics included ER, progesterone receptor (PR), and *ERBB2* (formerly *HER2* or *HER2/neu*) status. Treatments included surgical procedures and postsurgical chemotherapy, radiotherapy, and endocrine therapy.^27^ In addition, information on screening participation was extracted from the Stockholm mammographic screening data set,^28^ with screening data available for every screening round of each woman eligible for screening beginning in 1989. In this study, screening participation was defined as yes or no based on attendance at the most recent screening before the woman’s BC diagnosis.

Based on ER status, 2 additional subgroups were defined: ER positive and ER negative. Patient ER status was determined using immunohistochemical testing or radioimmunoassay, with cutoff values of more than 10% positive cells for immunohistochemical testing and more than 0 fmol/μg DNA for radioimmunoassay.^29^

### Family History of Breast Cancer

Family history of BC was the exposure of interest in this study and was defined as having at least 1 female FDR (mother, full sister, or daughter) diagnosed with BC at any time. Family members of each patient were identified through the Swedish Multi-Generation Registry,^26^ and the BC history of family members was obtained from the Swedish Cancer Registry.^24,30^ *International Classification of Diseases, Seventh Revision* codes (first 3 digits of 170) were used to identify BC; the Swedish Cancer Registry has close to 100% completeness for these codes.

Age at diagnosis of female FDR was further considered. Specifically, family history of BC was further categorized into having a family history of early-onset BC (defined as at least 1 female FDR diagnosed before age 40 years), having a family history of BC but not early-onset BC (defined as all female FDRs diagnosed after age 40 years), and having no family history of BC. Age 40 years is the commonly used cutoff for early-onset BC and was chosen based on previous research^16,17,18,19,20,21,22^ reporting that tumors diagnosed before age 40 years have a stronger genetic predisposition and distinct histopathological characteristics.

### Breast Cancer–Specific Mortality

Patients were followed up until they died of BC (outcome of interest), died of other causes, emigrated, reached age 85 years, or reached the end of follow-up on December 31, 2019. Deaths were identified from the Cause-of-Death Register,^31^ which is a high-quality, virtually complete register of all deaths in Sweden since 1952. Causes of death in this register were assessed by physicians following the process and rules for identifying causes of death, with the main underlying cause of death and other potential contributing causes defined for each record.^31^ In this study, BC-specific mortality was defined as BC being the main cause of death based on *International Statistical Classification of Diseases and Related Health Problems, Tenth Revision* code C50 or *International Classification of Diseases, Ninth Revision* code 174.

### Statistical Analysis

Flexible parametric survival models were fitted to investigate the association of family history with the outcome of BC-specific mortality. This model was chosen based on its flexibility, providing better model fit and more ease in modeling time-dependent covariates, which is an issue when the proportional hazards assumption is violated in the commonly used Cox proportional hazards model.^32^ Multivariable flexible models were built by adjusting for age at diagnosis, diagnosis year, number of female FDRs, tumor characteristics, and treatments received for the full cohort, ER-positive subgroup, and ER-negative subgroup. Furthermore, time-dependent hazard ratios (HRs) of family history were estimated in the multivariable model. Four degrees of freedom for main effects and 2 degrees of freedom for time-varying covariates were chosen based on Akaike information criteria. To improve model flexibility, continuous variables were transformed using restricted cubic splines, with the degree of freedom based on the lowest Akaike information criterion. Ten-year BC-specific survival by family history was also estimated and graphed from the fitted model.

We further conducted several sensitivity analyses to test the robustness of the findings. First, we restricted family history of BC among female FDRs to before the diagnosis of the index patient. Second, we used a cohort of patients diagnosed after 2007, when better data quality on ER status was found. Third, to check the stability of results, we restricted the analyses to diagnoses of sisters and mothers (excluding daughters) and only sisters (excluding mothers and daughters). Fourth, we conducted stratification analysis by tumor characteristic and diagnosis period to examine whether tumor characteristics or diagnosis period modified the association.

All analyses were performed using Stata MP software, version 17.0 (StataCorp LLC). User-written Stata software packages stpm2 and standsurv^32^ were used for the flexible parametric modeling. The significance threshold was 2-tailed *P* = .05.

## Results

### Population Characteristics and Events

Among 28 649 eligible patients, the mean (SD) age at BC diagnosis was 55.7 (10.4) years; 19 545 (68.2%) had ER-positive status, 4078 (14.2%) had ER-negative status, and 5026 (17.6%) had unknown ER status (Table 1). Among the full cohort, 5081 patients (17.7%) had at least 1 female FDR diagnosed with BC, and 384 (1.3%) had a family history of early-onset BC. Patient characteristics (age at diagnosis and menopausal status), screening participation, and tumor characteristics did not significantly differ between those with and without a family history of BC. During the follow-up period (median [IQR], 8.7 [4.1-15.1] years), 2748 patients (9.6%) died of BC, while 25 901 (90.4%) were censored because they died of other causes (n = 2745), emigrated (n = 300), reached age 85 years (n = 278), or remained at risk at the end of follow-up (n = 22 578).

**Table 1.** Baseline Patient and Tumor Characteristics Among Full Cohort and Estrogen Receptor Subgroups

Characteristic	Patients, No./total No. (%)^a^											
	Full cohort				ER-positive subgroup				ER-negative subgroup			
	Total (N = 28 649)	Family history of BC			Total (n = 19 545)	Family history of BC			Total (n = 4078)	Family history of BC		
		No (n = 23 568)	Yes (n = 5081)	*P* value		No (n = 16 085)	Yes (n = 3460)	*P* value		No (n = 3394)	Yes (n = 684)	*P* value
Age at diagnosis, mean (SD), y	55.7 (10.4)	55.7 (10.4)	55.8 (10.4)	.53	56.6 (10.4)	56.5 (10.4)	56.8 (10.2)	.11	53.1 (11.0)	53.2 (10.9)	52.9 (11.1)	.51
Menopause												
No	9699/28 405 (34.1)	7999/23 362 (34.2)	1700/5043 (33.7)	.12	6257/19 368 (32.3)	5190/15 935 (32.6)	1067/3433 (31.1)	.053	1610/4052 (39.7)	1336/3373 (39.6)	274/679 (40.4)	.58
Yes	17 543/28 405 (61.8)	14 432/23 362 (61.8)	3111/5043 (61.7)		12 366/19 368 (63.8)	10 152/15 935 (63.7)	2214/3433 (64.5)		2282/4052 (56.3)	1908/3373 (56.6)	374/679 (55.1)	
Unknown	1163/28 405 (4.1)	931/23 362 (4.0)	232/5043 (4.6)		745/19 368 (3.8)	593/15 935 (3.7)	152/3433 (4.4)		160/4052 (3.9)	129/3373 (3.8)	31/679 (4.6)	
Participation in screening^b^												
Yes	16 988/20 321 (83.6)	14 058/16 793 (83.7)	2930/3528 (83.0)	.33	12 042/14 289 (84.3)	9931/11 789 (84.2)	2111/2500 (84.4)	.80	2000/2567 (77.9)	1694/2158 (78.5)	306/409 (74.8)	.10
No	3333/20 321 (16.4)	2735/16 793 (16.3)	598/3528 (17.0)		2247/14 289 (15.7)	1858/11 789 (15.8)	389/2500 (15.6)		567/2567 (22.1)	464/2158 (21.5)	103/409 (25.2)	
Tumor size, mm												
<20	18 280/28 586 (63.9)	15 007/23 519 (63.8)	3273/5067 (64.6)	.064	12 502/19 533 (64.0)	10 295/16 076 (64.0)	2207/3457 (63.8)	.025	2026/4076 (49.7)	1672/3392 (49.3)	354/684 (51.8)	.50
20-50	8659/28 586 (30.3)	7122/23 519 (30.3)	1537/5067 (30.3)		6034/19 533 (30.9)	4931/16 076 (30.7)	1103/3457 (31.9)		1734/4076 (42.5)	1454/3392 (42.9)	280/684 (40.9)	
>50	1647/28 586 (5.8)	1390/23 519 (5.9)	257/5067 (5.1)		997/19 533 (5.1)	850/16 076 (5.3)	147/3457 (4.3)		316/4076 (7.8)	266/3392 (7.8)	50/684 (7.3)	
Tumor grade^c^												
1	3267/18 360 (17.8)	2698/15 213 (17.7)	569/3147 (18.1)	.48	3033/14 547 (20.8)	2492/12 032 (20.7)	541/2515 (21.5)	.67	45/2368 (1.9)	41/1995 (2.1)	4/373 (1.1)	.27
2	9067/18 360 (49.4)	7493/15 213 (49.3)	1574/3147 (50.0)		8059/14 547 (55.4)	6677/12 032 (55.5)	1382/2515 (55.0)		423/2368 (17.9)	349/1995 (17.5)	74/373 (19.8)	
3	6026/18 360 (32.8)	5022/15 213 (33.0)	1004/3147 (31.9)		3455/14 547 (23.8)	2863/12 032 (23.8)	592/2515 (23.5)		1900/2368 (80.2)	1605/1995 (80.5)	295/373 (79.1)	
Lymph node involvement												
No	17 595/25 807 (68.2)	14 471/21 216 (68.2)	3124/4591 (68.0)	.83	12 738/19 024 (67.0)	10 483/15 652 (67.0)	2255/3372 (66.9)	.91	2575/3959 (65.0)	2152/3293 (65.4)	423/666 (63.5)	.36
Yes	8212/25 807 (31.8)	6745/21 216 (31.8)	1467/4591 (32.0)		6286/19 024 (33.0)	5169/15 652 (33.0)	1117/3372 (33.1)		1384/3959 (35.0)	1141/3293 (34.6)	243/666 (36.5)	
ER status												
Positive	19 545/28 649 (68.2)	16 085/23 568 (68.2)	3460/5081 (68.1)	.07	NA	NA	NA	NA	NA	NA	NA	NA
Negative	4078/28 649 (14.2)	3394/23 568 (14.4)	684/5081 (13.5)		NA	NA	NA		NA	NA	NA	
Unknown	5026/28 649 (17.5)	4089/23 568 (17.3)	937/5081 (18.4)		NA	NA	NA		NA	NA	NA	
PR status												
Positive	16 577/23 447 (70.7)	13 612/19 333 (70.4)	2965/4114 (72.1)	.03	16 116/19 398 (83.1)	13 235/15 960 (82.9)	2881/3438 (83.8)	.22	459/4044 (11.4)	376/3369 (11.2)	83/675 (12.3)	.40
Negative	6870/23 447 (29.3)	5721/19 333 (29.6)	1149/4114 (27.9)		3282/19 398 (16.9)	2725/15 960 (17.1)	557/3438 (16.2)		3585/4044 (88.6)	2993/3369 (88.8)	592/675 (87.7)	
*ERBB2* status^d^												
Positive	2053/14 246 (14.4)	1765/11 852 (14.9)	288/2394 (12.0)	<.001	1364/12 221 (11.2)	1167/10 130 (11.5)	197/2091 (9.4)	.006	676/1990 (34.0)	587/1698 (34.6)	89/292 (30.5)	.17
Negative	12 193/14 246 (85.6)	10 087/11 852 (85.1)	2106/2394 (88.0)		10 857/12 221 (88.8)	8963/10 130 (88.5)	1894/2091 (90.6)		1314/1990 (66.0)	1111/1698 (65.4)	203/292 (69.5)	
Postsurgical chemotherapy												
No	15 882/24 566 (64.7)	13 056/20 195 (64.6)	2826/4371 (64.7)	>.99	10 861/16 735 (64.9)	8939/13 773 (64.9)	1922/2962 (64.9)	.99	1344/3509 (38.3)	1126/2917 (38.6)	218/592 (36.8)	.42
Yes	8684/24 566 (35.3)	7139/20 195 (35.4)	1545/4371 (35.3)		5874/16 735 (35.1)	4834/13 773 (35.1)	1040/2962 (35.1)		2165/3509 (61.7)	1791/2917 (61.4)	374/592 (63.2)	
Postsurgical radiotherapy												
No	5928/24 565 (24.1)	4795/20 194 (23.7)	1133/4371 (25.9)	.002	3298/16 734 (19.7)	2686/13 772 (19.5)	612/2962 (20.7)	.15	849/3509 (24.2)	688/2917 (23.6)	161/592 (27.2)	.06
Yes	18 637/24 565 (75.9)	15 399/20 194 (76.3)	3238/4371 (74.1)		13 436/16 734 (80.3)	11 086/13 772 (80.5)	2350/2962 (79.3)		2660/3509 (75.8)	2229/2917 (76.4)	431/592 (72.8)	
Postsurgical hormonal treatment												
No	7058/24 565 (28.7)	5818/20 194 (28.8)	1240/4371 (28.4)	.56	1267/16 734 (7.6)	1038/13 772 (7.5)	229/2962 (7.7)	.72	NA	NA	NA	NA
Yes	17 507/24 565 (71.3)	14 376/20 194 (71.2)	3131/4371 (71.6)		15 467/16 734 (92.4)	12 734/13 772 (92.5)	2733/2962 (92.3)		NA	NA	NA	

Abbreviations: BC, breast cancer; ER, estrogen receptor; NA, not applicable; PR, progesterone receptor.

^a^ The numbers of patients by categorical variable do not sum to the total number of patients in each group due to missing values; missing data were less than 18%, with the exception of tumor grade and *ERBB2* status, for which data were not available before 2004 and 2007, respectively.

^b^ Women aged 40 to 74 years were invited to attend mammographic screening at an interval of 18 or 24 months. Screening participation was defined as yes or no based on attendance at the most recent screening before breast cancer diagnosis.

^c^ Data were available beginning in 2004.

^d^ Data were available beginning in 2007.

### Family History of Breast Cancer and Breast Cancer–Specific Death

**Figure 2.**
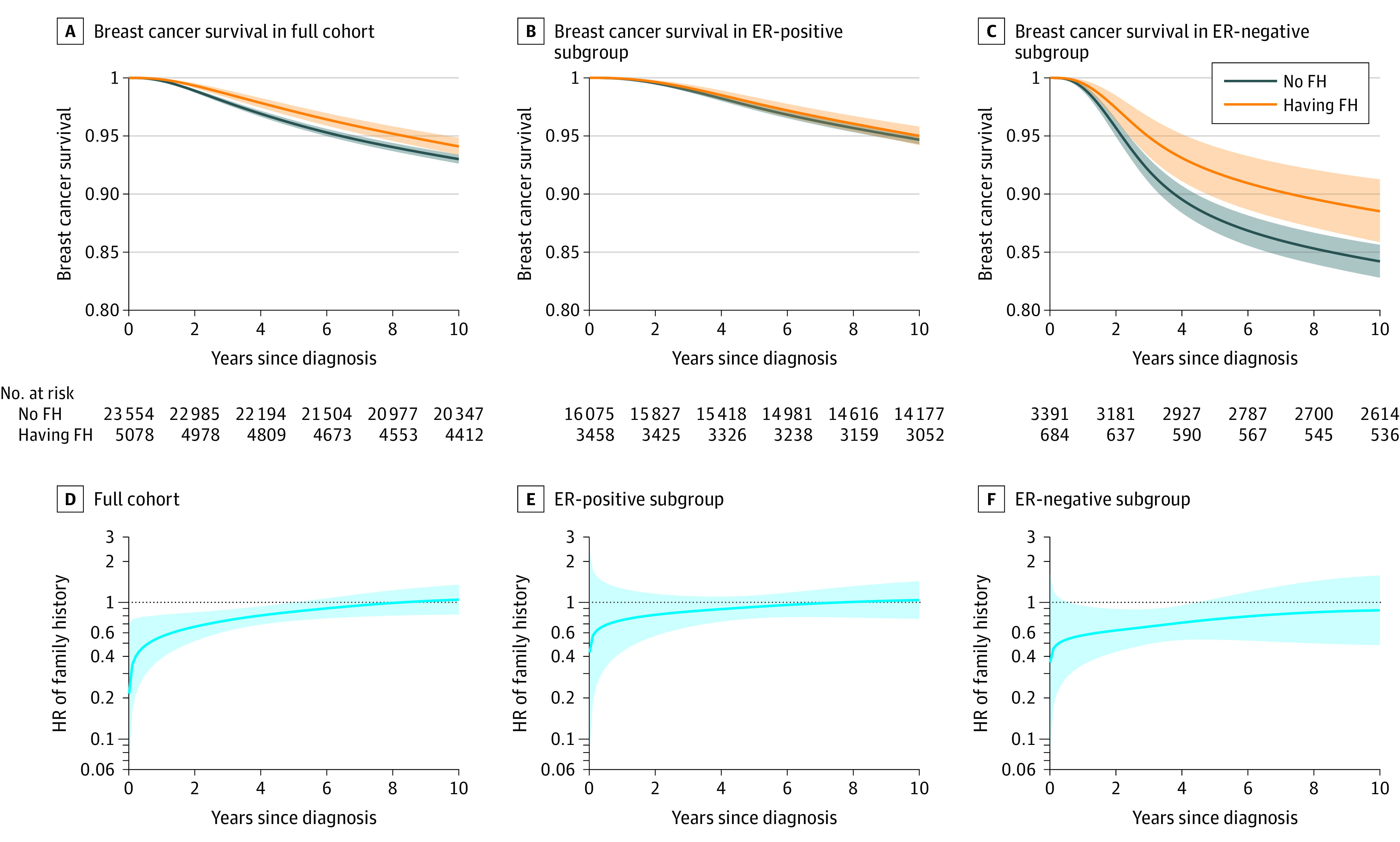
Survival by Family History (FH) of Breast Cancer and Time-Dependent Hazard Ratios (HRs) of Family History Among Full Cohort and Estrogen Receptor (ER) Subgroups Fully adjusted for age at diagnosis, year of diagnosis, number of female first-degree relatives, tumor size, lymph node status, receipt of chemotherapy, and receipt of radiotherapy. Shading indicates 95% CIs.

Among the full cohort, patients with a family history of BC had better BC-specific survival in the fully adjusted model (Figure 2A). Among the ER-positive subgroup, survival did not differ by family history, while among the ER-negative subgroup, better survival was observed in patients with a family history (Figure 2B and C).

The time-dependent HR of family history was lower than 1 during the first 5 years after diagnosis for the full cohort and the ER-negative subgroup, after which the HR decreased to nonsignificance (Figure 2D and F and Table 2). In the first 5 years, having a family history of BC was associated with a lower risk of BC-specific death among the full cohort (HR, 0.78; 95% CI, 0.65-0.95) and the ER-negative subgroup (HR, 0.57; 95% CI, 0.40-0.82). However, no association was found for the ER-positive subgroup (HR, 1.06; 95% CI, 0.80-1.40). In the second 5 years after BC diagnosis, none of the HRs for having family history was significant among the full cohort (HR, 1.03; 95% CI, 0.87-1.23), the ER-positive subgroup (HR, 1.05; 95% CI, 0.86-1.29), or the ER-negative subgroup (HR, 1.00; 95% CI, 0.65-1.53). All analyses of HR that were not adjusted for tumor characteristics and treatment resulted in similar estimates (eTable 1 in Supplement 1).

**Table 2.** Hazard Ratios for Breast Cancer–Specific Death Within the First and Second 5 Years by Presence or Absence of Family History Among Full Cohort and Estrogen Receptor Subgroups^a^

Interval	Full cohort			ER-positive subgroup			ER-negative subgroup		
	No. of deaths/No. of patients	HR (95% CI)	*P* value	No. of deaths/No. of patients	HR (95% CI)	*P* value	No. of deaths/No. of patients	HR (95% CI)	*P* value
**First 5 y**									
Family history									
No	1119/23 554	1 [Reference]	.02	470/16 075	1 [Reference]	.65	460/3391	1 [Reference]	.002
Yes	228/5078	0.78 (0.65-0.95)		104/3458	1.06 (0.80-1.40)		85/684	0.57 (0.40-0.82)	
**Second 5 y**									
Family history									
No	678/21 839	1 [Reference]	.33	459/15 299	1 [Reference]	.43	109/2846	1 [Reference]	.42
Yes	168/4742	1.03 (0.87-1.23)		102/3277	1.05 (0.86-1.29)		33/583	1.00 (0.65-1.53)	

Abbreviations: ER, estrogen receptor; HR, hazard ratio.

^a^ Fully adjusted for age at diagnosis, year of diagnosis, number of female first-degree relatives, tumor size, lymph node status, receipt of chemotherapy, and receipt of radiotherapy. In the full cohort model, ER status (positive, negative, or unknown) was further adjusted.

Sensitivity analyses restricted to female FDRs with BC diagnosis before the index patient’s diagnosis, the cohort diagnosed after 2007, or diagnoses of sisters and mothers had consistent results (eTable 2 in Supplement 1). Stratification analysis by tumor size, lymph node status, and diagnosis period suggested the findings were robust (eTable 3 in Supplement 1).

### Family History of Breast Cancer by Diagnosis Age and Mortality

**Figure 3.**
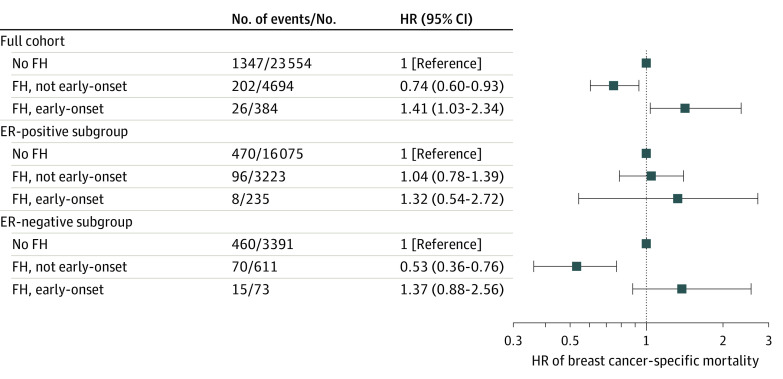
Hazard Ratios (HRs) for 5-Year Breast Cancer–Specific Death Among Patients by Family History (FH) of Breast Cancer and Early-Onset Breast Cancer Fully adjusted for age at diagnosis, year of diagnosis, number of female first-degree relatives, tumor size, lymph node status, receipt of chemotherapy, and receipt of radiotherapy. ER indicates estrogen receptor.

We further categorized family history by whether the female FDR was diagnosed with early-onset BC; the risk of BC-specific death by this family history category is shown in Figure 3. We report findings on 5-year mortality in this article because the association was limited to the first 5 years, with results for the second 5 years available in eTable 4 in Supplement 1. The finding of lower risk of BC-specific death at 5 years remained consistent for patients with a family history of non–early-onset BC (HR, 0.74; 95% CI, 0.60-0.93) (Figure 3). On the other hand, patients with at least 1 female FDR diagnosed with early-onset BC had a higher risk of BC-specific death (HR, 1.41; 95% CI, 1.03-2.34) compared with those without family history. Notably, patients with early-onset family history presented with more advanced tumor grades, and a higher proportion had ER-negative and PR-negative BC than those with no family history (eTable 5 in Supplement 1). In analyses of ER subgroups, a family history of non–early-onset BC was associated with a lower risk of death only in the ER-negative subgroup (HR, 0.53; 95% CI, 0.36-0.76).

## Discussion

This cohort study included, to our knowledge, one of the best-characterized cohorts of patients with BC to explore the association of family history with the prognosis of overall and ER-specific BC. We observed a lower risk of BC-specific death among patients with a family history of BC than those without. When investigating different ER subtypes, the association seemed to be mainly among the ER-negative subgroup. When further dividing family history by age of diagnosis of the female FDR, the lower risk remained for patients with non–early-onset family history but not for patients with early-onset family history.

Tumor characteristics are the most important factors in prognosis, and it is reasonable to assume that the better survival observed may be due to better tumor characteristics. Some previous studies^33,34,35^ have suggested that women with a family history of BC have greater willingness to attend screening and better access to health care resources,^33^ resulting in earlier detection with better tumor characteristics and lower BC mortality.^34,35^ However, we did not find that baseline tumor characteristics and participation rates at screening were significantly different among those with and without family history. This lack of difference was perhaps due to the well-established and long-standing mammographic screening program in Sweden, which has complete coverage by invitation and high attendance (80%) among the general population.^28,36^ This attendance rate is similar to those in the UK (81%), Netherlands (77%), and Finland (75%) and considerably higher than other countries in Europe, which have reported screening rates ranging from 13% to 70%.^36^ On the other hand, for women with early-onset family history, the adverse prognosis may be partly due to more aggressive tumors because these patients presented with more advanced tumor grades and a higher proportion of ER-negative and PR-negative tumors compared with those with no family history or family history of non–early-onset BC. However, after adjusting for tumor characteristics, the association between family history and lower BC mortality remained.

Health motivations and behaviors may provide 1 explanation for the better survival observed among patients with a family history of BC. Better access to health care resources (including familial BC clinics), higher motivation to treat among physicians and patients when patients have a family history, and better adherence to treatment are possible factors. In the Nurses’ Health Study,^37^ having a family history of BC or a self-reported history of benign breast diseases was reported to be positively associated with follow-up screening, which may result in earlier detection of recurrence. Other health-promoting behaviors included better weight management^38^ and exercise among women with a recent familial diagnosis of BC.^39^

It is interesting that we only found this survival benefit among patients with ER-negative but not ER-positive BC, although one would assume that better treatment adherence and health behaviors would also play a role in better survival for patients with ER-positive status. This finding could be due to the difference in disease natural history. For example, ER-positive BC normally has consistently low recurrence rates of approximately 1 in 10 patients within 5 years and 1 in 5 patients within 10 years, with better survival after relapse.^40^ In contrast, recurrence of ER-negative BC is typically rapid and occurs within 5 years after surgical treatment, with a higher mortality rate; thus, the cause of death can be specifically attributed to the disease itself.^40^ Because the association of family history with BC-specific mortality was observed to be limited to the first 5 years, the low recurrence and mortality rates of ER-positive BC within the first 5 years could be the reason an association was not found among patients with ER-positive status.

It is well known that family history of BC in FDRs is heterogeneous.^41^ In some cases, sporadic cancers are clustered in the same family due to shared family environment and lifestyle, given the high incidence of BC and shared lifestyle risk factors that could play a role in cancer occurrence; in other cases, hereditary BC with germline variants is passed down across generations.^42^ The biological characteristics and cancer prognoses differ in patients with different genetic variants, family history predispositions, and ages of onset.^43^ We observed that the subgroup of patients with early-onset family history had worse mortality, regardless of their ER status, than those without early-onset family history, which is consistent with studies finding worse survival in patients with early-onset BC themselves.^19,44^ The data from the present study can be used as a starting point for further studies addressing issues such as the cost-effectiveness of BC variant screening for all newly diagnosed patients who have early-onset family history; such screening would provide rich information to aid treatment and further research.

### Strengths and Limitations

This study has several strengths. First, to our knowledge, this study included one of the largest and most well-characterized cohorts of patients with BC, with good-quality data on defined ER subtypes. Data from multiple high-quality registries facilitated the possibility of complete follow-up of the BC outcomes. Second, the definition of family history is based on data linkage of a multigenerational registry and a cancer registry, resulting in almost 100% coverage and a low possibility of information bias. This family history definition is more robust compared with studies using self-reported family history. Third, this study was the first, to our knowledge, to include data on screening participation, and we found that participation did not differ by family history.

This study also has several limitations. First, ER status information started to be regularly collected in the 1990s, with a higher proportion of missing data in the beginning. However, this missing information is less of an issue because we selected a cohort of patients diagnosed since 1991. Given that missingness is a common issue with BC data, the missing values in this study were not higher than those found in a previous cohort study conducted in another setting.^45^ Second, some tumor characteristics were only available within the last 20 years, including tumor grade (since 2004) and *ERBB2* status (since 2007); thus, we did not include these characteristics in the main analysis. Because we observed different associations among ER subgroups, it might be of interest to investigate more detailed molecular subtypes in future research. Third, despite our large data set, when stratified by ER status, the wide CIs resulting from reduced power (particularly for ER-positive BC) bring uncertainty to the interpretation of higher risks associated with early-onset family history; thus, future studies with larger samples are needed to provide more precise estimates.

## Conclusions

This cohort study involving one of the largest cohorts of patients with BC to date found that those with a family history of BC did not necessarily have a worse prognosis. Patients with ER-negative BC and a family history of BC had more favorable outcomes in the first 5 years after diagnosis, possibly due to enhanced motivation to receive and adhere to treatment, which warrants further investigation. However, patients with a family history of early-onset BC had worse survival, suggesting that genetic testing of newly diagnosed patients with early-onset family history might provide useful information to aid treatment and future research.

## Notes

Supplement 1. **eTable 1.** Partly Adjusted Model Results on Family History and 5-Year Breast Cancer Mortality **eTable 2.** Sensitivity Analyses on Family History and 5-Year Breast Cancer–Specific Mortality **eTable 3.** Stratification Analysis: Hazard Ratios (HRs) and 95% CIs of Family Breast Cancer History on Breast Cancer–Specific Mortality Among Full Cohort, ER-Positive Subgroup, and ER-Negative Subgroup **eTable 4.** Hazard Ratios (HRs) of Family History and Early Onset on Breast Cancer–Specific Mortality Within the First and Second 5 Years Among Full Cohort and ER Subgroups **eTable 5.** Baseline Characteristics by Family History and Early Onset of First-Degree Female Relative

Supplement 2.
Data Sharing Statement
