# Supplementary material for: Analysis of Breast Cancer Family History, Estrogen Receptor Status, and Breast Cancer Outcomes in Sweden
Source: JAMA Netw Open. 2023 Jun 13;6(6):e2318053. doi: 10.1001/jamanetworkopen.2023.18053 (PMC10265300; doi:10.1001/jamanetworkopen.2023.18053)
Supplement: Supplement 1. — eTable 1. Partly Adjusted Model Results on Family History and 5-Year Breast Cancer Mortality eTable 2. Sensitivity Analyses on Family History and 5-Year Breast Cancer–Specific Mortality eTable 3. Stratification Analysis: Hazard Ratios (HRs) and 95% CIs of Family Breast Cancer History on Breast Cancer–Specific Mortality Among Full Cohort, ER-Positive Subgroup, and ER-Negative Subgroup eTable 4. Hazard Ratios (HRs) of Family History and Early Onset on Breast Cancer–Specific Mortality Within the First and Second 5 Years Among Full Cohort and ER Subgroups eTable 5. Baseline Characteristics by Family History and Early Onset of First-Degree Female Relative [file jamanetwopen-e2318053-s001.pdf]

Supplementary Online Content

Zhang Y, Wang QL, Zeng E, He W, Czene K. Analysis of breast cancer family history, estrogen receptor status, and breast cancer outcomes in Sweden. *JAMA Netw Open*. 2023;6(6):e2318053. doi:10.1001/jamanetworkopen.2023.18053

eTable 1. Partly Adjusted Model Results on Family History and 5-Year Breast Cancer Mortality

eTable 2. Sensitivity Analyses on Family History and 5-Year Breast Cancer–Specific Mortality

eTable 3. Stratification Analysis: Hazard Ratios (HRs) and 95% CIs of Family Breast Cancer History on Breast Cancer–Specific Mortality Among Full Cohort, ER-Positive Subgroup, and ER-Negative Subgroup

eTable 4. Hazard Ratios (HRs) of Family History and Early Onset on Breast Cancer–Specific Mortality Within the First and Second 5 Years Among Full Cohort and ER Subgroups

eTable 5. Baseline Characteristics by Family History and Early Onset of First-Degree Female Relative

This supplemental material has been provided by the authors to give readers additional information about their work.

© 2023 Zhang Y et al. *JAMA Network Open*.

eTable 1. Partly Adjusted Model Results on Family History and 5-Year Breast Cancer Mortality

**Population**	**Full cohort**				**ER-positive subgroup**				**ER-negative subgroup**			
	Event/N	HR	*p*	95% CI	Event/N	HR	*p*	95% CI	Event/N	HR	*p*	95% CI
Family history±												
No	1347/23554	1.00			470/16075	1.00			460/3391	1.00		
Yes	228/5078	**0.84**	**0.03***	0.72, 0.99	104/3458	1.01	0.90	0.80, 1.27	85/684	0.74	**0.03***	0.57, 0.97
Family history and early-onset±												
No	1119/23554	1.00			470/16075	1.00			460/3391	1.00		
Yes, not early onset	202/4694	**0.82**	**0.03***	0.69, 0.98	96/3223	1.04	0.74	0.81, 1.33	70/611	0.72	**0.02***	0.54, 0.95
Yes, early onset	26/384	**1.56**	**0.03***	1.03, 2.37	8/235	1.41	0.33	0.70, 2.86	15/73	1.55	0.12	0.88, 2.72

±Multivariable flexible parametric survival model, adjusted for age at diagnosis, year of diagnosis and number of female first-degree relatives;

© 2023 Zhang Y et al. *JAMA Network Open*.

eTable 2. Sensitivity Analyses on Family History and 5-Year Breast Cancer–Specific Mortality

Population	Full cohort				ER-positive subgroup				ER-negative subgroup			
	Event/N	HR	p-value	95% CI	Event/N	HR	p-value	95% CI	Event/N	HR	p-value	95% CI
Only using family history before the diagnosis of index person												
History of BC among all female FDR												
No	1073/25322	1			462/17183	1			442/3667	1		
Yes	109/3327	0.82	0.098	0.65, 1.03	54/2362	1.01	0.91	0.74, 1.38	37/411	**0.65**	**0.029***	0.44, 0.95
Using a clean cohort between 2007-2015[±]												
History of BC among sisters and mother[#]												
No	409/7042	1			254/5429	1			139/911	1		
Yes	102/1822	0.82	0.22	0.61, 1.11	71/1420	1.10	0.57	0.78,1.55	24/205	**0.39**	**0.019***	0.18, 0.85
History of BC among sisters[^]												
No	279/4593	1			181/3553	1			90/580	1		
Yes	47/768	0.80	0.35	0.50, 1.27	32/596	1.02	0.91	0.60, 1.73	12/96	**0.32**	**0.059**	0.10, 1.04

[±] The clean cohort was chosen of those diagnosed after 2007, when a better data quality on ER status was reported, and before 2015 to ensure a minimum 5-year follow-up until the end of 2019.
[^] patients who have at least one sister were included
[#] patients who have at least one sister or mother were included
Fully adjusted for age at diagnosis, year of diagnosis, number of female FDR of interest, tumor pathology size, lymph node status, chemotherapy and radiotherapy.

© 2023 Zhang Y et al. *JAMA Network Open*.

eTable 3. Stratification Analysis: Hazard Ratios (HRs) and 95% CIs of Family Breast Cancer History on Breast Cancer–Specific Mortality Among Full Cohort, ER-Positive Subgroup, and ER-Negative Subgroup

Stratification		**Full cohort**	**ER-positive subgroup**	**ER-negative subgroup**
By tumor size				
<20mm	FH: No	1.00	1.00	1.00
	FH: Yes	**0.60 (0.41, 0.88)**	0.97 (0.58, 1.60)	**0.34 (0.18, 0.67)**
>=20mm	FH: No	1.00	1.00	1.00
	FH: Yes	0.84 (0.67, 1.05)	0.98 (0.73, 1.33)	**0.72 (0.47, 1.00)**
By lymph node				
No	FH: No	1.00	1.00	1.00
	FH: Yes	**0.65 (0.46, 0.93**)	0.75 (0.45, 1.25)	**0.56 (0.32, 0.99)**
Yes	FH: No	1.00	1.00	1.00
	FH: Yes	0.88 (0.70, 1.11)	1.17 (0.86, 1.57)	**0.66 (0.44, 0.98)**
By diagnosis period				
1991-1998	FH: No	1.00	1.00	1.00
	FH: Yes	**0.64 (0.43, 0.96)**	0.82 (0.46, 1.46)	**0.57 (0.31, 1.07)**
1999-2006	FH: No	1.00	1.00	1.00
	FH: Yes	0.84 (0.67, 1.05)	0.98 (0.73, 1.33)	**0.72 (0.47, 1.00)**
2007 onwards	FH: No	1.00	1.00	1.00
	FH: Yes	0.76 (0.56,1.02)	0.95 (0.65, 1.39)	**0.57 (0.33, 0.96)**

HR, hazard ratio; CI, confidence interval; ER+, estrogen receptor positive; ER-, estrogen receptor negative; FH, family history (among female first-degree relatives)
Bold type indicates statistical significance using p value<0.05
Fully adjusted for age at diagnosis, year of diagnosis, number of female first-degree relatives, tumor pathology size, lymph node status, chemotherapy and radiotherapy, excluding the stratification variable for each stratification analysis. Additionally in the full cohort model, ER status (ER+, ER-, or ER status unknown) is further adjusted

© 2023 Zhang Y et al. *JAMA Network Open*.

eTable 4. Hazard Ratios (HRs) of Family History and Early Onset on Breast Cancer–Specific Mortality Within the First and Second 5 Years Among Full Cohort and ER Subgroups

**Population**	**Full cohort**			**ER-positive subgroup**			**ER-negative subgroup**		
	Event/N	HR (95% CI)	*p*	Event/N	HR (95% CI)	*p*	Event/N	HR (95% CI)	*p*
First 5 years									
Family history									
No	1347/23554	1.00		470/16075	1.00		460/3391	1.00	
FH, early-onset	26/384	**1.41 (1.03,2.34)**	**0.03**	8/235	1.32 (0.54,2.72)	0.53	15/73	1.37 (0.88, 2.56)	0.12
FH, not early-onset	202/4694	**0.74 (0.60,0.93)**	**0.01**	96/3223	1.04 (0.78,1.39)	0.74	70/611	**0.53 (0.36, 0.76)**	**<0.001**
Second 5 years									
Family history									
No	678/21839	1.00		459/15299	1.00		109/2846	1.00	
FH, early-onset	11/346	0.91 (0.47, 1.76)	0.77	6/221	0.71 (0.29, 1.72)	0.45	4/55	1.86 (0.58, 5.93)	0.29
FH, not early-onset	157/4396	0.89 (0.72, 1.09)	0.27	96/3056	0.82 (0.29, 1.72)	0.15	29/528	1.02 (0.64, 1.64)	0.90

Fully adjusted for age at diagnosis, year of diagnosis, number of female first-degree relatives, tumor pathology size, lymph node status, chemotherapy and radiotherapy. Additionally in the full cohort model, ER status (ER-positive, ER-negative, or ER status unknown) is further adjusted.

© 2023 Zhang Y et al. *JAMA Network Open*.

eTable 5. Baseline Characteristics by Family History and Early Onset of First-Degree Female Relative

	Total	Family history and Early-onset of FDR			
		No	Yes, Early-onset	Yes, no early-onset	p-value
	N=28649	N=23568	N=384	N=4697	
Age at diagnosis	55.69 (10.41)	55.67 (10.41)	52.76 (12.12)	56.02 (10.20)	<0.001
Menopause					<0.001
No	9699 (34.1%)	7999 (34.2%)	170 (44.5%)	1530 (32.8%)	
Yes	17543 (61.8%)	14432 (61.8%)	200 (52.4%)	2911 (62.5%)	
Unknown	1163 (4.1%)	931 (4.0%)	12 (3.1%)	220 (4.7%)	
Participation in screeening					0.33
Yes	16988 (83.6%)	14058 (83.7%)	184 (80.3%)	2746 (83.2%)	
No	3333 (16.4%)	2735 (16.3%)	45 (19.7%)	553 (16.8%)	
Tumor pathology size					0.079
0-20mm	18280 (63.9%)	15007 (63.8%)	261 (68.1%)	3012 (64.3%)	
20-50mm	8659 (30.3%)	7122 (30.3%)	108 (28.2%)	1429 (30.5%)	
>=50mm	1647 (5.8%)	1390 (5.9%)	14 (3.7%)	243 (5.2%)	
Lymph node metastasis					0.90
No	17595 (68.2%)	14471 (68.2%)	224 (67.1%)	2900 (68.1%)	
Yes	8212 (31.8%)	6745 (31.8%)	110 (32.9%)	1357 (31.9%)	
Tumor grade					0.086
1	3267 (17.8%)	2698 (17.7%)	36 (16.1%)	533 (18.2%)	
2	9067 (49.4%)	7493 (49.3%)	99 (44.2%)	1475 (50.5%)	
3	6026 (32.8%)	5022 (33.0%)	89 (39.7%)	915 (31.3%)	
ER					<0.001
Positive	19545 (82.7%)	16085 (82.6%)	235 (76.3%)	3225 (84.1%)	
Negative	4078 (17.3%)	3394 (17.4%)	73 (23.7%)	611 (15.9%)	
PR					0.006
Positive	16577 (70.7%)	13612 (70.4%)	202 (66.0%)	2763 (72.6%)	
Negative	6870 (29.3%)	5721 (29.6%)	104 (34.0%)	1045 (27.4%)	
HER2					<0.001
Positive	2053 (14.4%)	1765 (14.9%)	10 (6.2%)	278 (12.4%)	
Negative	12193 (85.6%)	10087 (85.1%)	150 (93.8%)	1956 (87.6%)	
Post-surgery chemotherapy					0.28
No	15882 (64.7%)	13056 (64.6%)	202 (60.7%)	2624 (65.0%)	
Yes	8684 (35.3%)	7139 (35.4%)	131 (39.3%)	1414 (35.0%)	
Post-surgery radiotherapy					0.009
No	5928 (24.1%)	4795 (23.7%)	88 (26.4%)	1045 (25.9%)	
Yes	18637 (75.9%)	15399 (76.3%)	245 (73.6%)	2993 (74.1%)	
Post-surgery endocrine therapy					0.12
No	7058 (28.7%)	5818 (28.8%)	110 (33.0%)	1130 (28.0%)	
Yes	17507 (71.3%)	14376 (71.2%)	223 (67.0%)	2908 (72.0%)	

Number of patients by categorical variables may not sum up to total number of patients due to missing values, missing all below 18% except tumor grade and HER2 that started to be collected at a later time.

© 2023 Zhang Y et al. *JAMA Network Open*.
