# Supplementary material for: Analysis of Breast Cancer Family History, Estrogen Receptor Status, and Breast Cancer Outcomes in Sweden
Source: JAMA Netw Open. 2023 Jun 13;6(6):e2318053. doi: 10.1001/jamanetworkopen.2023.18053 (PMC10265300; doi:10.1001/jamanetworkopen.2023.18053)
Supplement: Supplement 2. — Data Sharing Statement [file jamanetwopen-e2318053-s002.pdf]

Data Sharing Statement

Zhang. Analysis of Breast Cancer Family History, Estrogen Receptor Status, and Breast Cancer Outcomes in Sweden. *JAMA Netw Open*. Published June 13, 2023. doi:10.1001/jamanetworkopen.2023.18053

Data
Data available: Yes
Data types: Data dictionary
How to access data: Data dictionary is available on request to the corresponding author, and some can also be found at https://karmastudy.org/contact/data-access/data-sources/.
When available: With publication

Supporting Documents
Document types: None

Additional Information
Who can access the data: For researchers whose proposed use of the data has been approved
Types of analyses: For research-related purpose
Mechanisms of data availability: With a signed data access agreement
